# Defect minimized Ag-ZnO microneedles for photocatalysis

**DOI:** 10.1007/s11356-020-09433-5

**Published:** 2020-06-23

**Authors:** Sanjay Gopal Ullattil, M. J. Jabeen Fatima, Ahmed Abdel-Wahab

**Affiliations:** 1 Chemical Engineering Program, Texas A&M University at Qatar, 23874, Education City, Doha, Qatar; 2 Department of Nanoscience and Technology, University of Calicut, Malappuram, Kerala 673635 India

**Keywords:** Heavy Ag loading, Ag-ZnO microneedles, Surface plasmon, Electron density, Solar energy, Photocatalysis

## Abstract

A facile solution processing strategy has been developed for the formation of Ag-modified ZnO microneedles at various calcination temperatures such as 300, 500, and 700 °C (AZ3, AZ5, and AZ7 respectively). Due to the heavy doping of AgNO_3_, Ag^+^ ions have been incorporated in to the crystal lattice of ZnO in all the Ag-ZnO samples, which facilitated the formation of Ag-ZnO microneedle morphology with minimized defect states, and obviously, the plasmon peaks were observed due to Ag modification. These Ag-ZnO microneedle structures have been evaluated for their photocatalytic performance using methylene blue as model target contaminant and their activity was compared with the commercially available titania P25 photocatalyst. The photoactivity of all the Ag-ZnO microneedle structures was significantly higher than that of the commercially available P25 photocatalyst with the most active Ag-ZnO material having a photocatalytic activity ~ 1.4 times greater than that of P25 titania.

**Electronic supplementary material:**

The online version of this article (10.1007/s11356-020-09433-5) contains supplementary material, which is available to authorized users.

## Introduction

ZnO has been widely used as a photocatalyst in its pure form or after modification by the incorporation of foreign materials such as metals (Vaiano et al. 2018), non-metals (Kumari et al. 2019), and organics (Ansari et al. 2013). ZnO is a semiconductor with a large band gap of 3.37 eV. It is a suitable candidate in photocatalysis due to its ecofriendly nature, low cost, and high catalytic efficiency (Ong et al. 2018). When a photon energy of higher than or equal to its band gap energy is irradiated onto ZnO, the electrons in the valance band (VB) jump to the conduction band (CB) leading to the formation of electron-hole pairs (Ong et al. 2018). These electron-hole pairs can recombine within nanoseconds, which limits the photocatalytic efficiency of ZnO material. This unfavorable situation can be overcome by introducing a noble metal-semiconductor interface that can generate a Schottky barrier effect to inhibit the electron-hole recombination and thereby increase the photocatalytic efficiency (Yan et al. 2014).

Silver is one of the prominent candidates that can be used as dopant/modifier which generate Ag-loaded ZnO nanostructures (Ag-ZnO) with a lower Fermi level (*E*_f_) than the CB of ZnO (Ren et al. 2010). Thus, the photogenerated electron transfers to the *E*_f_ of Ag-ZnO allowing Ag to act as a sink for electrons promote the interfacial charge transfer kinetics between Ag and ZnO, and reduce the electron-hole recombination, which results in enhancing the photocatalytic efficiency. Also, Ag can originate surface plasmons and increase the absorption wavelength, thereby enhancing the visible light photocatalytic activity (Kavitha et al. 2019).

The morphology of the photocatalyst surface plays an important role in its photoactivity. We have previously developed a solution processing strategy for the synthesis of ZnO microrods (Ullattil et al. 2016) which has been adopted in this study for Ag doping (10 mol%) on ZnO to fabricate Ag-ZnO microneedles at various high temperatures. AgNO_3_ that was used as the source of Ag has its ability to act as a morphological sharpener. The presented method is very suitable for industrial production of Ag-ZnO microneedles as photocatalysts. An elongated prismatic growth can be favored by zinc precipitation using NH_4_OH and this has been well-documented (Ullattil et al. 2016). The influence of zinc counterions on the shape evolution (Govender et al. 2004) and the pH dependence of ZnO and Zn(OH)_2_ formation are also available (Zhao et al. 2006). The present work was designed and planned to exploit the following features of an Ag-modified ZnO such as (1) Zn(OH)_2_ has been suggested as a prerequisite for the controlled growth of ZnO needles (Ullattil et al. 2016; McBride et al. 2003), (2) the lower Fermi level of Ag as compared with the CB of ZnO (Ren et al. 2010), and (3) strong ability of Ag to form a Ag-ZnO interface and to generate a Schottky barrier for minimizing the e^−^–h^+^ recombination (Yan et al. 2014). In addition, heavy loading of AgNO_3_ is employed for Ag incorporation into the ZnO crystal lattice rather than forming a surface linkage between them.

## Materials and methods

### Materials

Zinc nitrate hexahydrate 98% (Sigma-Aldrich), ammonium hydroxide (30%, Merck), nitric acid (72%, Merck), and silver nitrate, ACS reagent, ≥ 99% (Sigma-Aldrich) were purchased and were used as received. The dye used for photocatalytic study was methylene blue (Aldrich Chemicals) and was used without further purification. Deionized water was used in all the experiments.

### Preparation of Ag-modified ZnO

In a typical experiment, zinc nitrate (18.6 g, 0.25 M) was dissolved in 250 mL of water. The solution was stirred and ammonium hydroxide was slowly added drop by drop until pH reaches a value of 8 to ensure complete precipitation of white zinc hydroxide. After stirring for 2 h, the so-formed zinc hydroxide solid was filtered using a Buchner apparatus. The precipitate was washed until it was free from ammonium and nitrate ions. The washed precipitate was dispersed in 300 mL of water and stirred continuously for 3 h. At the same time, 10% nitric acid was added drop by drop to prevent aggregation of particles until the solution pH reached pH 6 (Ullattil et al. 2016). For silver modification, 0.025 M silver nitrate (1.061 g in 250 mL of water) was added to the above suspension and stirred continuously again for 6 h. The suspension was allowed to dry at 100 °C. The so-formed Ag-modified ZnO precursor (AZ) was calcined at high temperatures 300, 500, and 700 °C for 2 h at a temperature ramp rate of 5 °C min^−1^ followed by heating at the highest temperature and is herein after termed as AZ3, AZ5, and AZ7 respectively.

### Characterization

The crystallinity of Ag-modified ZnO powders was characterized by X-ray diffraction (XRD) technique with Miniflex 600 X-ray diffractometer in the diffraction angle range 2*θ* = 20–80° using Cu Kα radiation. The average crystallite size “*φ*” of the samples was estimated using Scherrer’s equation (Eq. 1), by measuring the line broadening of (101) main intensity peak, where *λ* is the wavelength of Cu Kα radiation (*λ* = 1.5401 nm), *β* is the full width at half-maximum, and *θ* is Bragg’s angle (Cullity and Stock 2001). Surface morphologies were examined by using Hitachi-Su6600 field emission scanning electron microscope. The formation of ZnO was further confirmed by measuring Fourier transform infrared spectroscopy (FTIR) spectra of Ag-ZnO samples using Jasco-FT/IR-4100 spectrophotometer in the range of 4000–400 cm^−1^. The Raman and photoluminescence spectra were performed using Thermo Fischer Raman spectrophotometer and Perkin Elmer LS 45 spectrophotometer. Photocatalysis of all the samples were studied using Xe lamp (450 W) as simulated solar light attached with Scientech power control. The distance from the light source to the solution was set to 18 cm and the intensity was measured using a Global solar power instrument and the measured intensity was 100 mW cm^−2^. Both the optical measurements and photocatalytic studies were recorded with Jasco-V-550-UV/VIS spectrophotometer.

1 $$ \varphi =\frac{0.9\ \lambda }{\beta\ \mathrm{Cos}\theta } $$

### Photocatalysis

The powdered sample (0.1 g) was dispersed in 50 mL of methylene blue dye solution of concentration 10^−5^ M and stirred in the dark for 4 h to eliminate the possibility for adsorption during the photocatalytic reaction and thus maintaining the adsorption-desorption equilibrium. Then, the solution was irradiated under 1 sun with continuous stirring. Samples were collected in each 2 min and centrifuged for 5 min at 3000 rpm to separate solid particles, followed by the absorption spectra measurements using a UV spectrophotometer.

## Results and discussion

### Mechanism

Zn(OH)_2_ is a prerequisite for the controlled growth of ZnO needles (McBride et al. 2003) and an elongated prismatic growth can be favored by Zn(OH)_2_ precipitation by additions of NH_4_OH to solutions containing Zn^2+^ which has also been reported (Ullattil et al). In this study, zinc nitrate was dissolved in water as Zn^2+^ and NO_3_^−^ and Zn^2+^ ions precipitated as Zn(OH)_2_ after the addition of NH_4_OH base. The addition of HNO_3_ to Zn(OH)_2_-dispersed solution favored a H^+^ ion templating around the precipitated Zn(OH)_2_ particles. The pH of the solution was adjusted to pH 6. The Ag^+^ as well as additional amounts of NO_3_^−^ was supplied upon the addition of AgNO_3_ to the solution of pH 6. According to the phase stability diagrams for the ZnO-H_2_O and Zn(OH)_2_-H_2_O systems at 298 K and the thermodynamic data, the soluble species such as Zn^2+^ and ZnOH^+^ are stable below pH 7 (Padmanabhan et al. 2009). These structures on to the oxygen-rich face of the Ag-incorporated ZnO nuclei occur to form microrod particles having an outermost Zn^2+^ layer at its one end. Such particles then grow to form the microneedles by a heterogeneous nucleation-assisted growth among the Ag-incorporated ZnO/Zn(OH)_2_ species. The consumption of more NO_3_^−^ species upon AgNO_3_ addition and calcination at high temperatures results in the elimination of the growth barrier that in turn dictates the tapering of one end of the particles to form pointed tips, hence microneedles. These morphological evolutions of all the Ag-modified ZnO samples were followed using scanning electron microscopy (SEM), which led us to some interesting observations regarding the microneedle formation.

### Scanning electron microscopy

**Fig. 1:**
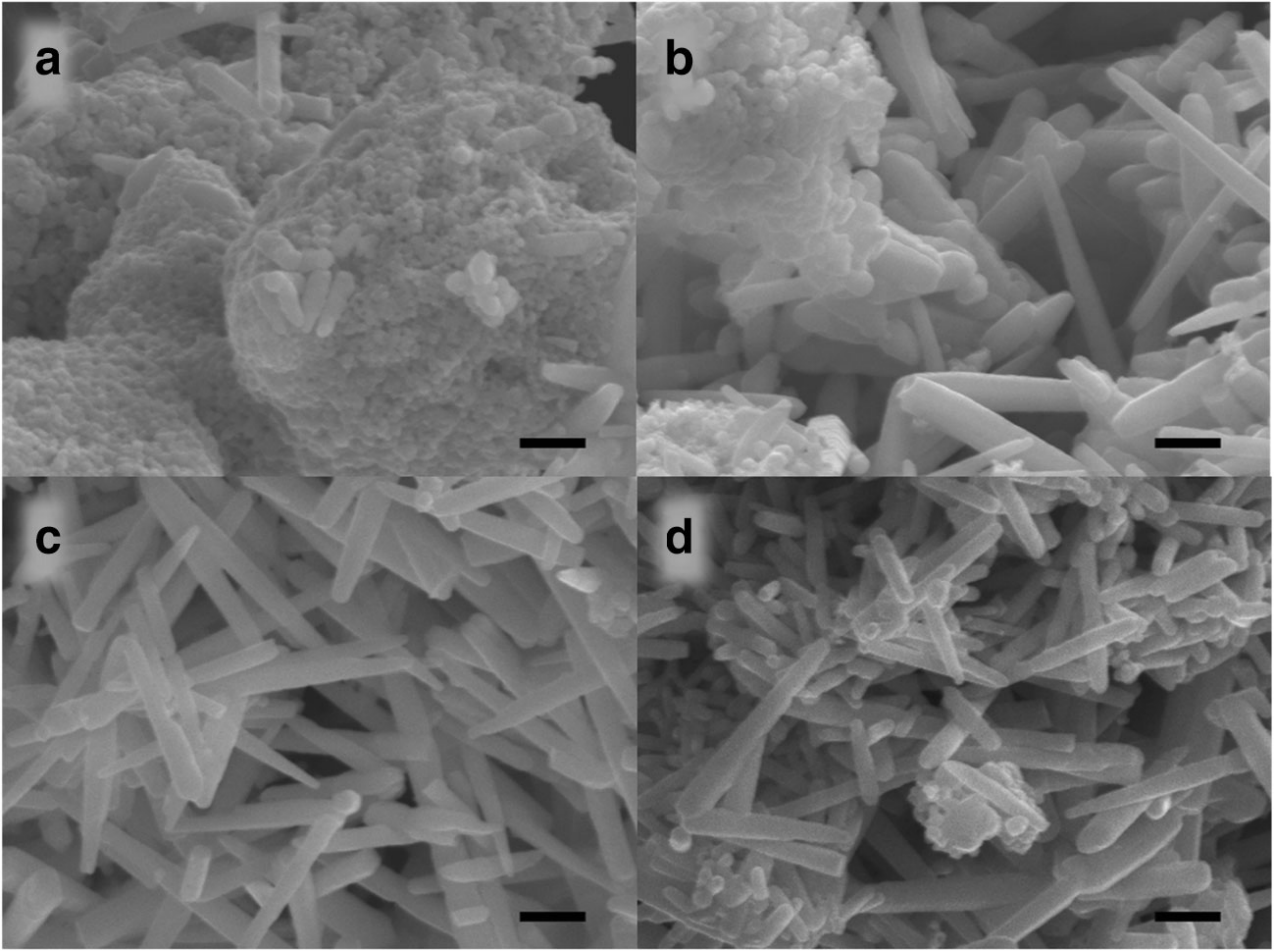
SEM image of Ag-ZnO microneedles: **a** AZ, **b** AZ3, **c** AZ5, and **d** AZ7 (scale bar is 1 μm)

The micrographs show that the precursor (AZ) has no specific morphology although it tends to form rod morphology even at 100 °C as shown in Fig. 1a. As the calcination temperature increased to 300 °C (AZ3), needles are also formed along with rods and thus the growth of microneedles is confirmed (Fig. 1b). While looking into AZ5, uniform distribution of microneedles is observed (Fig. 1c) which could facilitate high degree of photocatalysis. The uniform distribution of microneedles has been destroyed at 700 °C. As it can be seen in Fig. 1d, the SEM of AZ7, the pointed tips of the needles almost disappeared and tend to aggregate. This is due to the densification of ZnO normally observed at high temperatures (Mazaheri et al. 2008). Ag doping led the way to the formation of needle-like structures which are formed from ZnO microrods; i.e., AgNO_3_ is working as a sharpener of the microrods, and so in the present context, AgNO_3_ acts here as a “structural sharpener.”

### X-ray diffraction

**Fig. 2:**
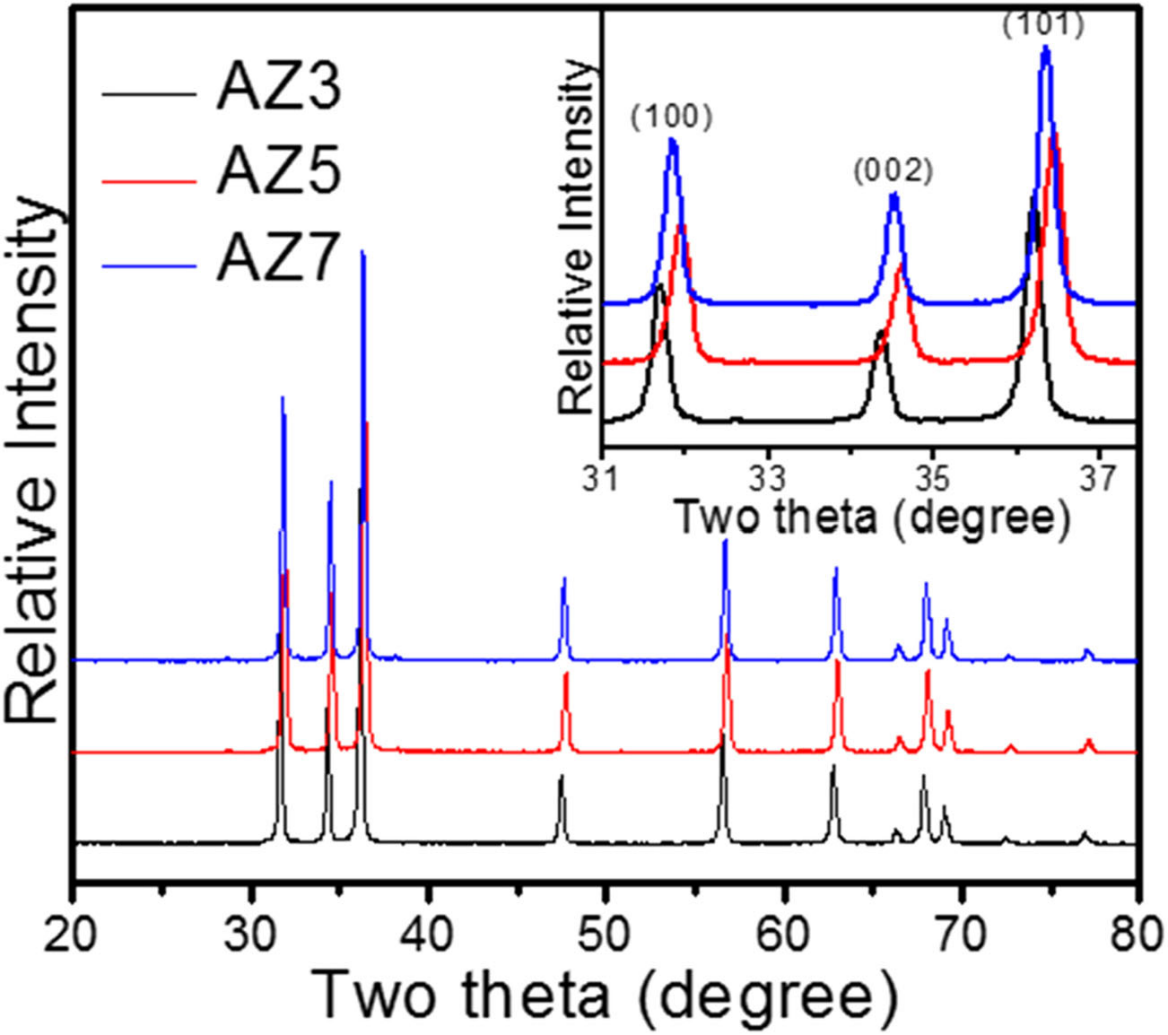
XRD of Ag-ZnO microneedles—AZ3, AZ5, and AZ7, inset zoom out XRD peaks from 31 to 38°

The XRD patterns of AZ3, AZ5, and AZ7 are shown in Fig. 2, and they show good agreement with the hexagonal wurtzite ZnO (JCPDS file no. 36-1451) (Kuriakose et al. 2014). It is also noticed that, as the calcination temperature increases, remarkable peak shift is observed in the XRD pattern (inset of Fig. 2) which is a clear confirmation of the incorporation of Ag^+^ into the crystal lattice of ZnO (Liu et al. 2017). The incorporation of Ag^+^ ions into the Zn^2+^ sites is too difficult to achieve because of the large ionic radius difference between them (126 pm for Ag^+^ and 74 pm for Zn^2+^) (Ahmad et al. 2013). Since the ionic radius of Ag^+^ is far greater than that of Zn^2+^, it can be confirmed that Ag^+^ possessed the interstitial position of ZnO crystal lattice. The average crystallite sizes obtained from XRD were 42.7, 34.3, and 43.4 nm respectively for AZ3, AZ5, and AZ7. It is worth noting that the amorphous ZnO precursor approaches crystallinity even at 100 °C after Ag incorporation (Fig. S1) (Zhao et al. 2006). From this figure, it is clear that Ag doping has minimized the amorphous peaks which were present in bare ZnO. Interestingly, the most intense peak of ZnO precursor is a (300) peak which could be assigned to Zn(OH)_2_, whereas for the Ag-ZnO precursor, the most intense peak orientation is along the (100) plane which corresponds to the nanocrystalline ZnO (Pimentel et al. 2014). Thus, it can be confirmed that AgNO_3_ is not only a useful dopant and structural sharpener but also a phase purifier.

### Fourier transform infrared spectroscopy

**Fig. 3:**
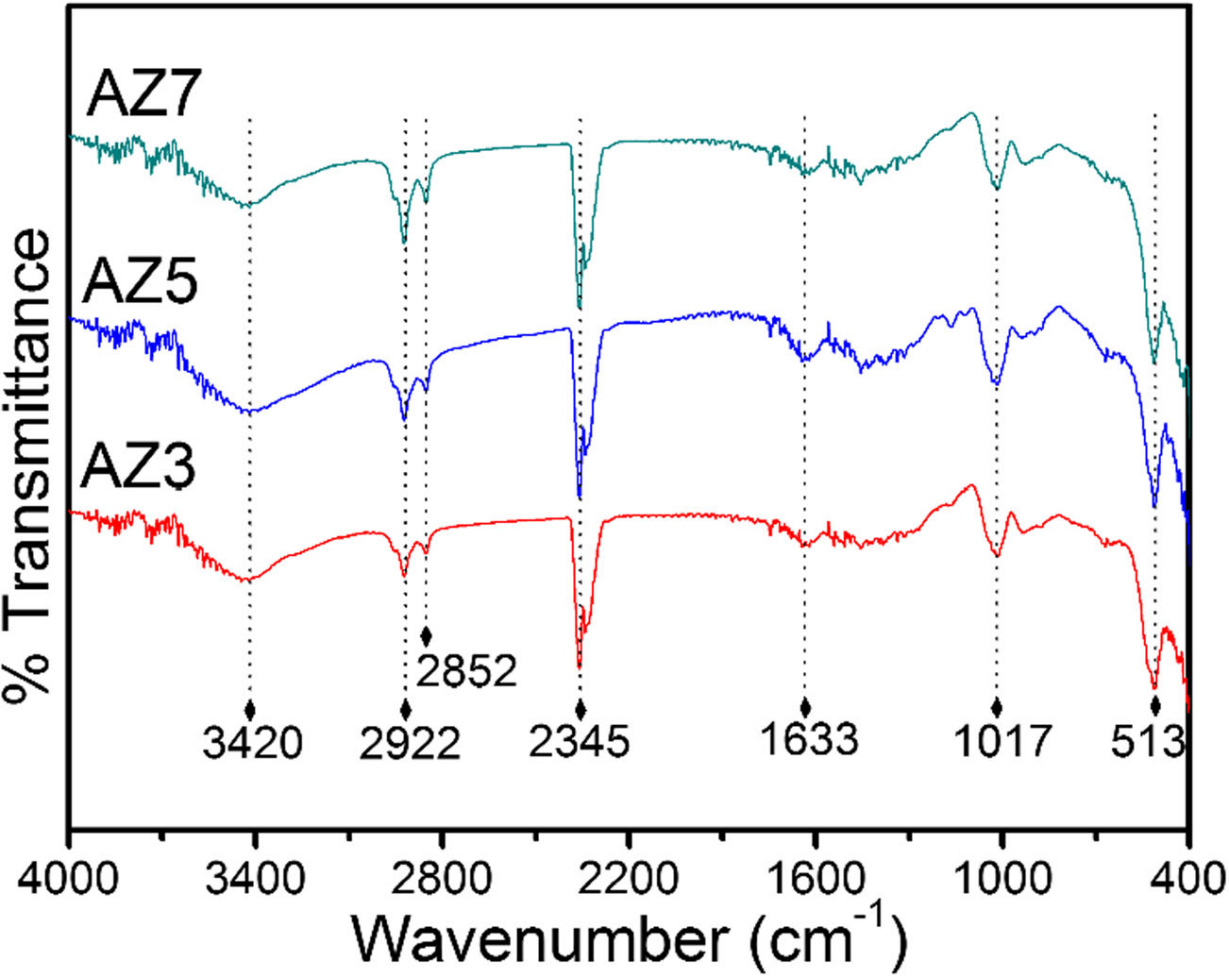
FTIR spectra of Ag-ZnO micro needles—AZ3, AZ5, and AZ7

FTIR spectra were recorded to further confirm the formation of Ag-modified ZnO. From FTIR studies (Fig. 3), the broad band at ~ 513 cm^−1^ is attributed to Zn-O stretching vibration (Saravanan et al. 2011). The peaks at 3420 and 1633 cm^−1^ correspond to the stretching and bending vibrations of surface-adsorbed –OH and water molecules (Moussawi and Patra 2016). Even though Ag^+^ ions have been incorporated into the crystal lattice of ZnO, no peaks are observed as characteristics of direct bonding between Zn and Ag. Thus, it is argued that the Ag^+^ ions that have been incorporated into the lattice sites possess the interstitial space of the ZnO structure. For all the samples, a band at 1017 cm^−1^ is present, which is ascribed to the threshold frequency band representing the activation energy for electronic conduction which is beneficial for the high photocatalytic activity (Jimenez-Gonzalez et al. 1998). This threshold energy band that was dominant in AZ5 also enhances the photocatalytic efficiency of AZ5 as compared with other samples, which will be discussed in detail under the Photocatalysis section. A high intense peak at ~ 2354 cm^−1^ is due to surface-adsorbed –CO_2_ molecules (Saber et al. 2017) and as a consequence, the peaks at 2922 and 2852 cm^−1^ can be attributed to C-OH peaks originated due to the interaction of CO_2_ with weakly adsorbed –OH groups (Saber et al. 2017). The E1(LO) mode of ZnO microneedles has also been shown in Fig. S2 and the band positions were found around 512 cm^−1^. According to previous report of Zheng et al., the E1(LO) mode should shift with the change in the density of oxygen vacancies (Zheng et al. 2007). In addition, the precursor (AZ) has an E1(LO) mode of vibration at ~ 554 cm^−1^ whereas for all the other samples, they have bands at lower wave number at ~ 512 cm^−1^ indicating the low amount of oxygen vacancy defects in all the samples except AZ. In other words, the defect states are more in the ZnO precursor and the defect states are highly reduced when calcined at 300–700 °C.

### UV-visible absorption spectra and Tauc plots

**Fig. 4:**
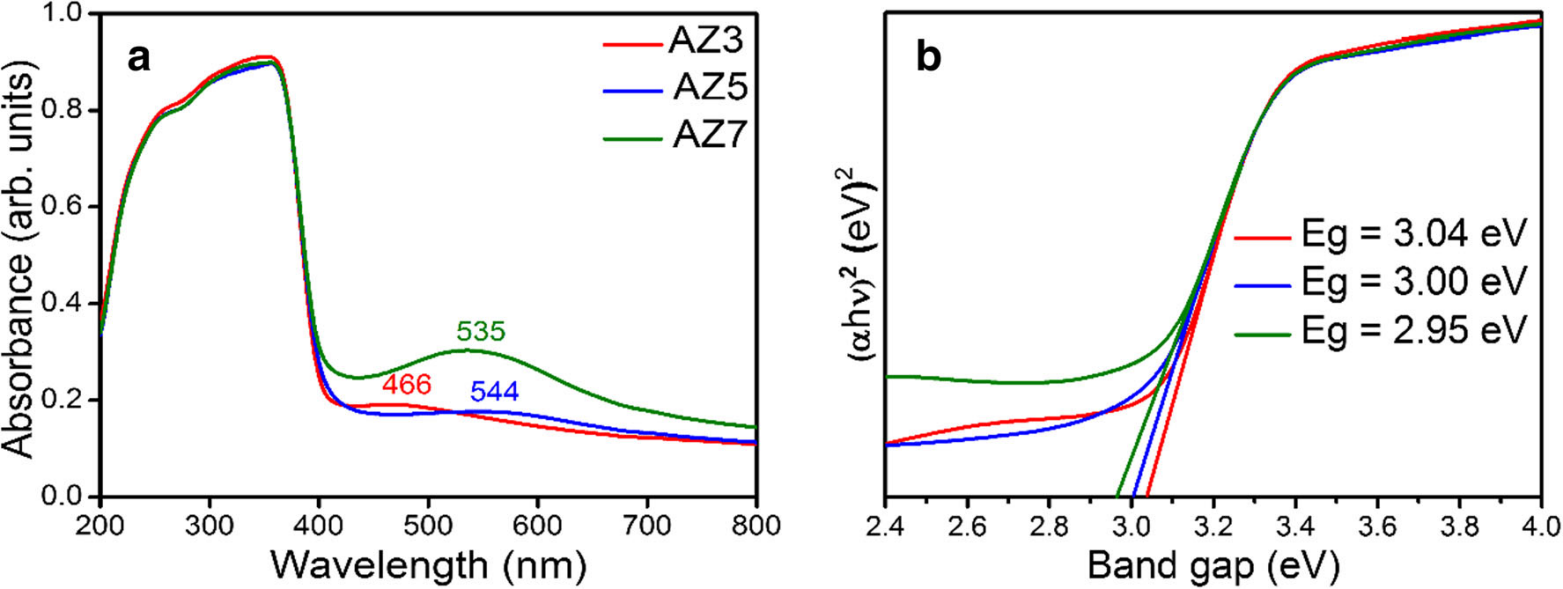
**a** UV-visible spectra of Ag-ZnO needles—AZ3, AZ5, and AZ7—and **b** Tauc plot of Ag-ZnO microneedles—AZ3, AZ5, and AZ7

The absorption spectra of Ag-ZnO samples are presented in Fig. 4a. As the figure indicates, with the increase in temperature, surface plasmon resonance peak shift occurs. Generally, surface plasmon peaks for Ag are observed at the wavelength range of 430–460 nm. Here, for Ag-ZnO precursor, the plasmon peak is observed at 463 nm and as the temperature increased to 300 °C, the peak red shifted to 466 cm^−1^. Interestingly at 500 °C, the surface plasmon peak is red shifted to a large extent and the value is at 544 cm^−1^. But as the temperature reached to 700 °C, a blue shift in plasmon peak was observed having a value of 535 cm^−1^. The plasmon absorption (*λ*_p_) of silver can be represented as Eq. 2 (Chen et al. 2011)

2 $$ {\lambda}_{\mathrm{p}}={\left[\left(4\ \pi 2c2{m}_{\mathrm{eff}}{\varepsilon}_{\mathrm{o}}\right)/ Ne2\right]}^{\frac{1}{2}} $$

where *m*_eff_ is the effective mass of the free electron of the metal and *N* is the electron density. As the equation suggests, the position of surface plasmon peak shifts to higher wavelength region; i.e., red shift occurs when the electron density of the metal is very low. Thus, it can be confirmed that, as the temperature increases, the electron density decreases up to 500 °C. But when the temperature was raised to 700 °C, the electron density started increasing which is confirmed by the blue shift of the surface plasmon peak occurred from 544 to 535 cm^−1^. Here, the Ag modification paved the way to increased electron density and the same metal acts as the sink or collector of those electrons which tend to recombine with the holes present at the surface of the CB of ZnO. Therefore, the recombination can be minimized and improve photocatalysis. The Tauc plot of all the calcined samples are also shown in Fig. 4b, where the band gap energy slightly decreased as the calcination temperature increased and the values observed are 3.04, 3.00, and 2.95 eV for AZ3, AZ5, and AZ7 respectively.

### Raman spectroscopy

**Fig. 5:**
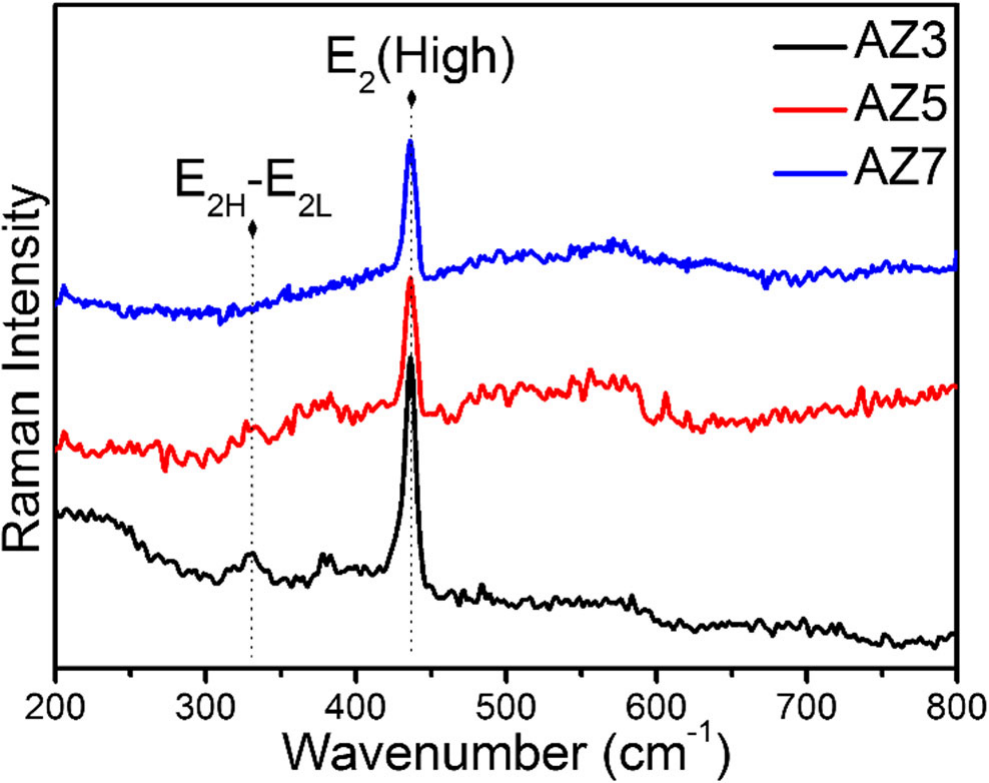
Raman spectra of Ag-ZnO microneedles—AZ3, AZ5, and AZ7

To further confirm the crystallinity of the synthesized solids, Raman spectroscopy was used and the spectra are displayed in Fig. 5. All the Ag-ZnO samples show a sharp peak at ~ 440 cm^−1^ corresponding to the E_2_ (high) mode of the Raman active mode which is the main characteristic of wurtzite hexagonal phase of ZnO (Ngo-Duc et al. 2012). Generally, a peak of E_1_ (LO) mode around 580 cm^−1^ is observed for ZnO having oxygen vacancy defect states and no such peak is visible for any of the samples, which reveals the low concentration of oxygen vacancy states and higher crystalline nature of the materials (Khosravi-Gandomani et al. 2014). This finding is in agreement with the E1 (LO) mode of the samples obtained from FTIR. In addition, AZ3 and AZ5 shows the peak at ~ 331 cm^−1^ ascribed to *E*_2H_-*E*_2L_ (multi phonon process mode) which is absent in AZ7 indicating that both of these samples show single crystalline nature but AZ7 does not (Umar and Hahn 2006).

### Photoluminescence spectroscopy

**Fig. 6:**
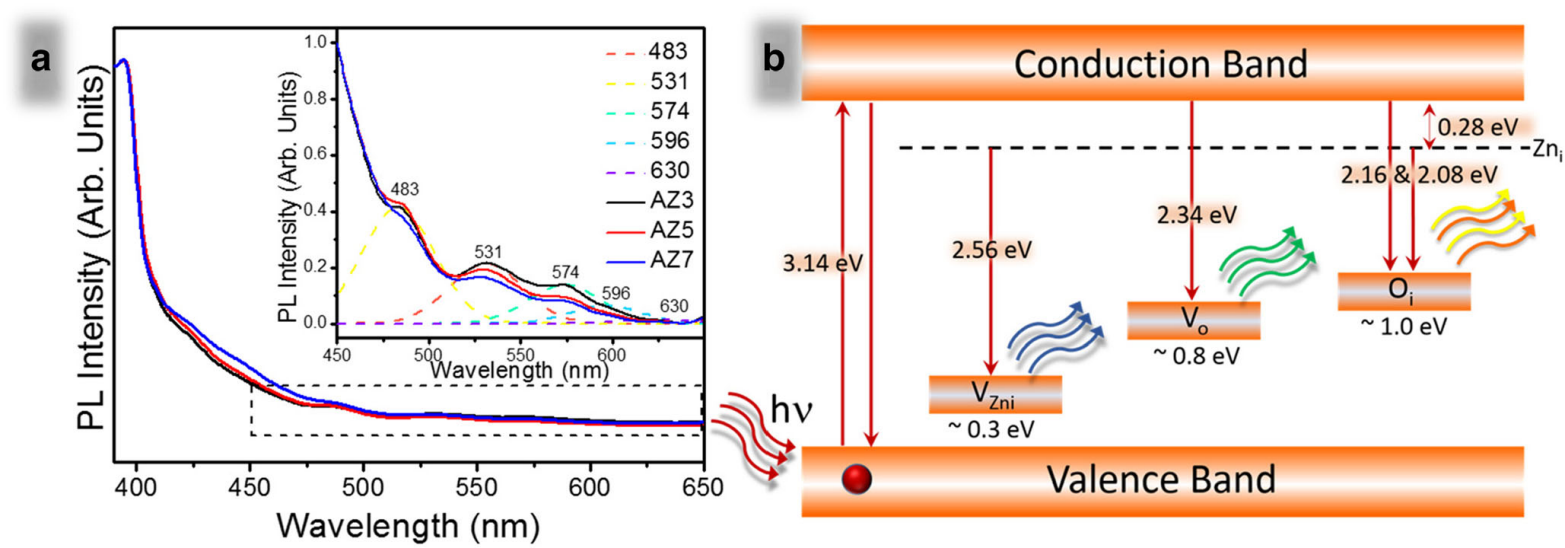
**a** PL spectra of Ag-ZnO microneedles—AZ3, AZ5, and AZ7—and **b** schematic representation of emission of photons from various defect states

The photoluminescence spectra of the Ag-doped samples are shown in Fig. 6a. In the inset of the figure, the deconvoluted spectra of the photoluminescence spectroscopy (PL) in the range 450–650 nm fitted using the Gaussian fit method (to differentiate the peaks) are given in order to differentiate the deep level emissions of the doped samples. The spectra generally indicate lower defect state after doping. In-depth investigation of the spectra reveals that the band edge emission is observed at 394 nm in the UV region and is almost equivalent to 3.14 eV. The convolution resulted in the peaks obtained at 483, 531, 574, 596, and 630 nm. The peak at 483 (2.56 eV) is assigned to electron transition from zinc vacancy level observed at 0.28 eV from conduction band level to single ionized zinc vacancies (2.84 eV) resulting a blue emission spectra (Lin et al. 2001). A green emission is observed at 531 (2.34 eV) nm due to electron transition from the conduction band to the oxygen ion vacancies (Vo) situated at ~ 0.8 eV from the valence band. As oxygen ion vacancies are lower, the peak is almost negligible in the spectrum (Amiruddin and Kumar 2014a, b). The peak at 574 (2.16 eV) and 596 (2.08 eV) resulting in a yellow and orange emission respectively is probably due to transition of electrons from the conduction band and zinc ion vacancy to the oxygen interstitials (Oi) located at ~ 1 eV (Amiruddin and Kumar 2014a, b). Red emissions at 630 nm are negligibly small and hence are almost invisible indicating deep level emissions are almost nil. The overall PL spectrum indicates minimum defect states in the zinc oxide after doping with silver. A schematic representation of the emission is given in Fig. 6b. These results regarding the defect states are in good agreement with the results obtained from E1 (LO) mode of FTIR and the Raman spectra.

### Photocatalysis

**Fig. 7:**
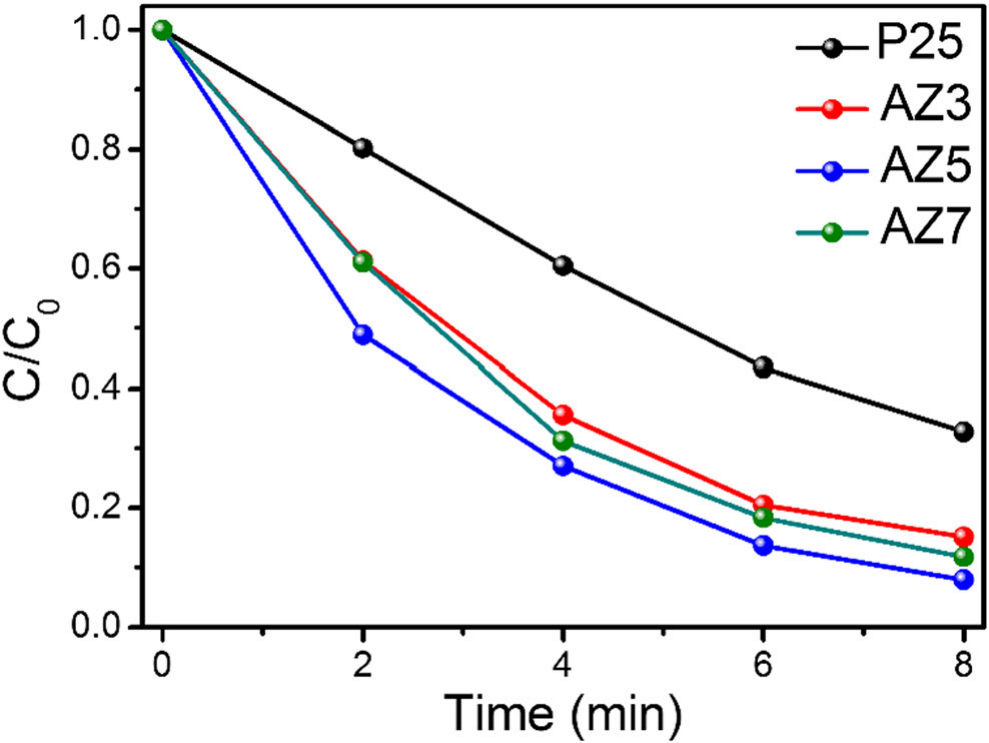
Photodegradation kinetics of MB under 1 sun using P25, AZ3, AZ5, and AZ7

Methylene blue (MB) is adopted as the representative organic pollutant to evaluate the photocatalytic performance of Ag-ZnO microneedles. Commercially available photocatalyst P25 titania has also been evaluated and thus used as a reference photocatalyst. The photocatalytic activities of the as-prepared Ag-modified sample at different temperatures and P25 are shown in Fig. 7. *C*_0_ and *C* are the initial concentration after reaching the adsorption equilibrium and the measured concentration at the corresponding reaction time of MB respectively. As seen in Fig. 7, the degradation of MB over the Ag-ZnO microneedles exhibits higher photocatalytic activity compared with commercially available P25 titania photocatalyst. The degradation of MB over P25, AZ3, AZ5, and AZ7 catalysts was monitored over 8 min and the maximum photoactivity was observed for AZ5. The degradation rate percentage (*x*) in Eq. 3 was 84.8, 92.1, and 88.2 for AZ3, AZ5, and AZ7 respectively, whereas the photodegradation rate percentage of P25 was 67.2 after 8 min. The order of photoactivity was AZ5 > AZ7 > AZ3 > P25 and it can be seen that all the photocatalytic processes here followed the first order kinetics (Fig. S4).

3 $$ x=\frac{C_0-C}{C_0}\times 100 $$

The Fermi level originated due to Ag modification has restrained the electron-hole recombination during photocatalysis and thus, Ag acts as a sink of electrons. Here, for all the samples, the activation energy for electronic conduction is high which is evident from FTIR. The corresponding band is slightly higher in intensity for AZ5 and hence, high photocatalytic activity is observed. In addition to this, the microneedle morphology, surface plasmon effect, and single crystalline nature were collectively beneficial for the high photoactivity of all the samples. The observed electron density (*N*) was highest for AZ3 and the order can be represented as *N*_AZ5_ < *N*_AZ7_ < *N*_AZ3_. Obviously, the order of effective mass of free electrons would be the reverse and can be represented as *N*_AZ5_ > *N*_AZ7_ > *N*_AZ3_. Generally, the higher effective mass of free electrons leads to reduced photocatalytic performance. However, here, the maximum efficiency is observed for AZ5 which has the maximum effective mass of electrons. If a large difference between the effective masses of holes (*m*_h+_) and electrons (*m*_e−)_ occur, the condition may be reversed. The relative ratio of effective masses (*D*) can be explained as Eq. 4 (Faraji et al. 2015)

4 $$ D={m}_{\mathrm{h}+}/{m}_{\mathrm{e}-} $$

If the *D* value is higher, the electron mobility would increase and thus, the recombination of the photoinduced charges can be restrained (Opoku et al. 2017). So, it is expected that the effective mass of holes is greater for all the samples and therefore, the order of the relative ratio of effective masses (*D*) as well as the photocatalytic efficiency follows the order AZ5 > AZ7 > AZ3.

## Conclusions

A modified solution processing strategy has been developed for the synthesis of Ag-modified ZnO microneedles with minimized defect states. The Ag-modified ZnO precursor was calcined at 300, 500, and 700 °C. The facile incorporation of Ag^+^ into the ZnO led the way to minimization of the amorphous peaks that in bare ZnO precursor synthesized at 100 °C. In addition to that, the morphology of all the Ag-modified ZnO samples is microneedles. Absorption spectra proved the high visible light absorption of Ag-modified ZnO samples, which has extended the wavelength cutoff toward higher wavelength visible region. The “sharpening effect” of AgNO_3_ was confirmed by SEM analysis, which has resulted in the morphological conversion of microrods to needles. These materials were employed as photocatalysts for the photodegradation of methylene blue under 1 sun solar illumination. The photoactivity followed the order of AZ5 > AZ7 > AZ3 > P25, where the percentage of photodegradation for the most active performer AZ5 and P25 was 92.1 and 67.2 after 8 min of 1 sun solar illumination. It is expected that the efficiency of these materials can be exploited in photocatalytic water splitting and other solar energy applications.

## Electronic supplementary material

ESM 1 (PDF 95 kb)
