# Supplementary material for: Defect minimized Ag-ZnO microneedles for photocatalysis
Source: Environ Sci Pollut Res Int. 2020 Jun 23;27(29):37036–43. doi: 10.1007/s11356-020-09433-5 (PMC7456407; doi:10.1007/s11356-020-09433-5)
Supplement: Supplementary file 1 — (PDF 95 kb) [file 11356_2020_9433_MOESM1_ESM.pdf]

Supplementary Material

Defect Minimized Ag-ZnO Micro-needles for Photocatalysis

Sanjay Gopal Ullattil,[a]* Jabeen Fatima M J,[b] Ahmed Abdel-Wahab[a]*

[a] *Department of Chemical Engineering, Texas A&M University, Education City, Doha, Qatar*

[b] *Department of Nanoscience and Technology, University of Calicut, Kerala, India-673635*

*sanjay.ullattil@qatar.tamu.edu;ahmed.abdel-wahab@qatar.tamu.edu

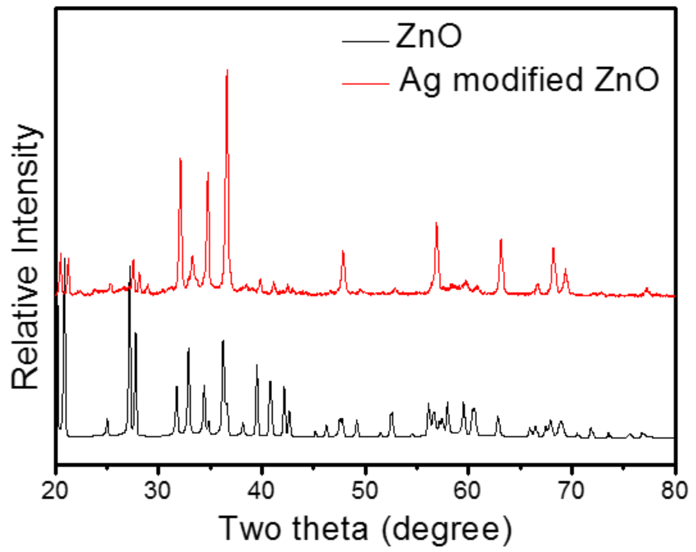

Figure S1. XRD of ZnO microrods (reprinted with permission from American Chemical Society-Ref.9)

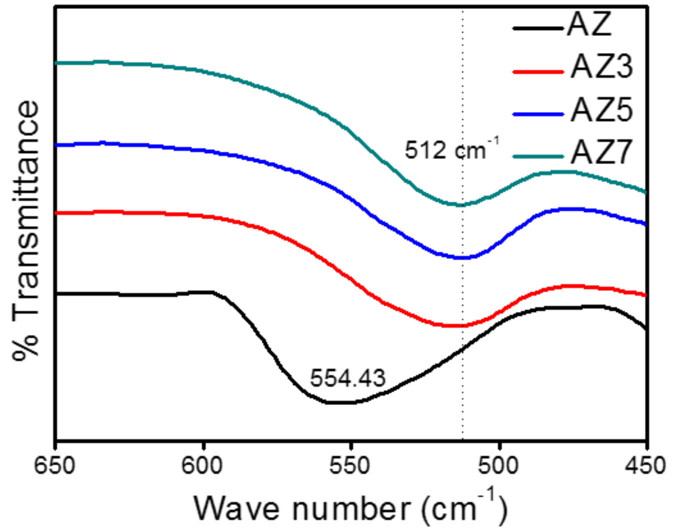

Figure S2. E1 (LO) mode of vibration of AZ, AZ3, AZ5 and AZ7

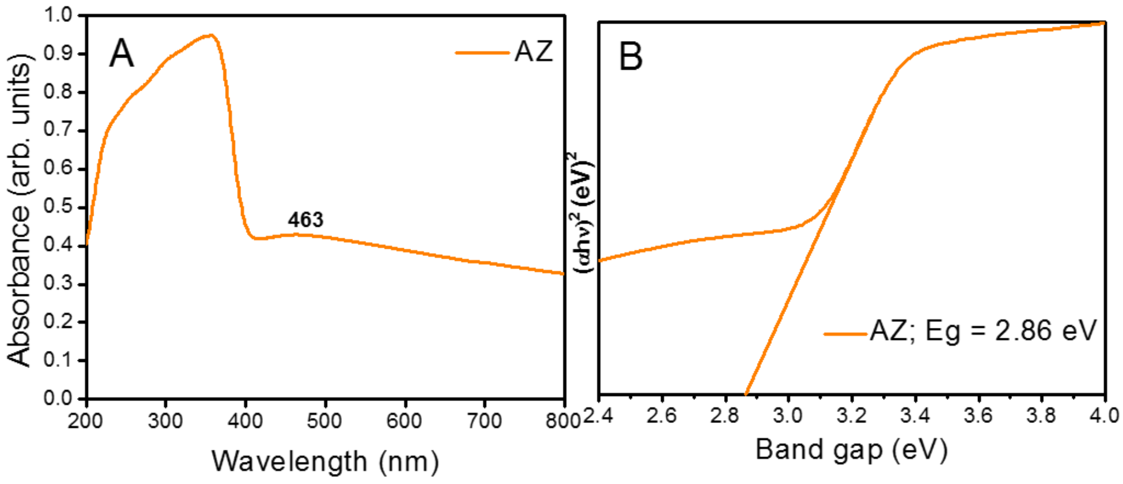

Figure S3. A)UV-Vis Spectrum and B) Tauc Plot of AZ

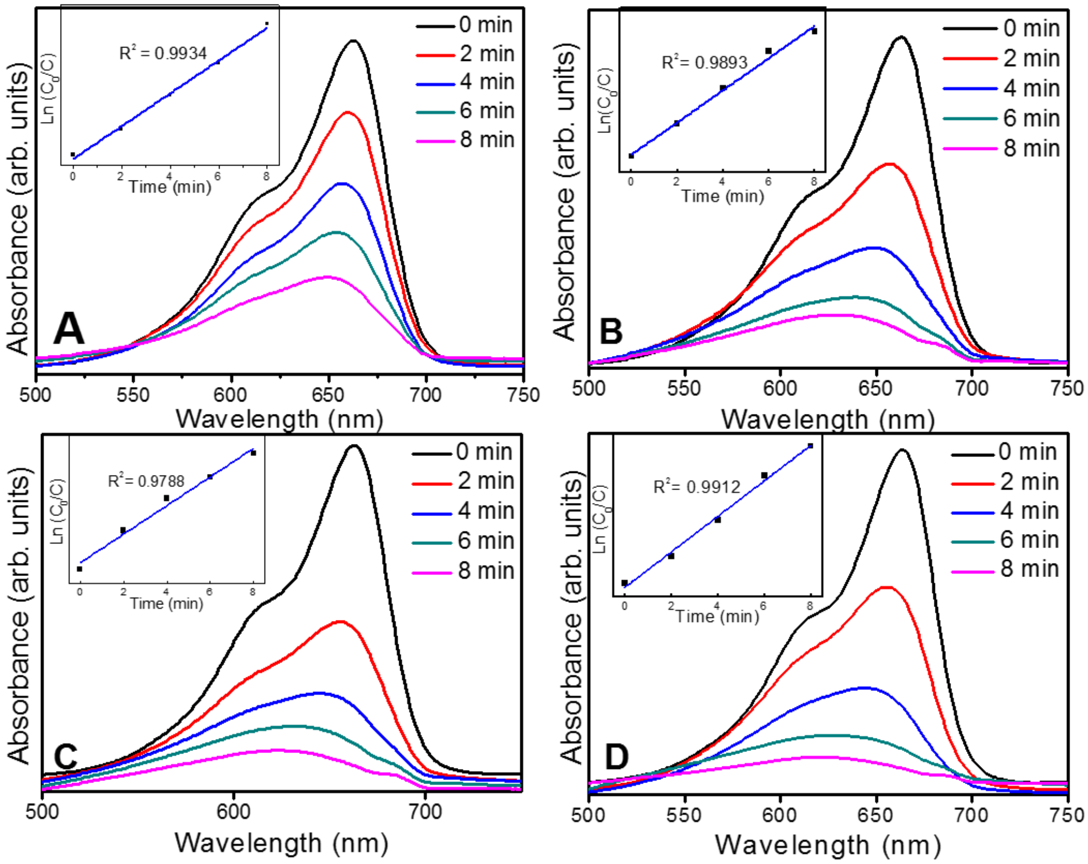

Figure S4. UV-Visible spectra of MB photodegradation and corresponding kinetic plot A) P25, B) AZ3 C) AZ5 and D) AZ7
